# Divergent directions of bias: affective forecasting in younger vs. older adults and the role of affective working memory

**DOI:** 10.3389/fpsyg.2026.1843889

**Published:** 2026-07-16

**Authors:** Yangdongping Feng, Di Lu

**Affiliations:** School of Medical Humanities, China Medical University, Shenyang, Liaoning, China

**Keywords:** affective forecasting, affective working memory, aging, overestimation, underestimation

## Abstract

**Background:**

Affective forecasting is crucial for decision-making. Age-related differences in forecasting accuracy are documented, but the underlying cognitive mechanisms, such as affective working memory (AWM), remain unclear. This study examined whether AWM capacity moderates age differences in affective forecasting accuracy.

**Methods:**

We recruited younger and older adults to complete an affective forecasting task. Participants forecasted their emotional responses to positive and negative events based on verbal descriptions, and reported their actual emotions 1 week later upon viewing related images. AWM was assessed using an affect maintenance task. We employed linear mixed models (LMMs) with random intercepts for participants to account for repeated measures (forecast and experience), examining the effects of age group, event valence, and AWM on forecasting accuracy (bias score). Cluster-bootstrap confidence intervals (1,000 resamples) were computed for simple effects to obtain robust interval estimates.

**Results:**

Both age groups showed significant affective forecasting bias, but the pattern differed: older adults underestimated future positive emotions, whereas younger adults overestimated them. As predicted, AWM capacity was lower in older adults. However, contrary to our hypothesis, AWM did not significantly moderate the relationship between age and forecasting accuracy. The observed age differences in bias direction were not explained by variations in AWM.

**Conclusions:**

The findings confirm that age-related positivity effects manifest differently in affective forecasting, leading to divergent bias patterns. Importantly, the AWM decline in aging does not directly account for these differences in forecasting accuracy. This suggests that other cognitive or motivational factors may be more central. The results highlight the need to look beyond single-mechanism explanations when designing interventions to improve emotional prediction and decision-making in later life.

## 1 Introduction

Affective forecasting, the ability to predict one's future emotions, is crucial for health-related decision-making across the lifespan. For older adults, however, biases in this process (such as over- or under-estimating emotional outcomes) pose a distinct public health challenge. These biases can undermine critical decisions about adherence to medical regimens, health behaviors, and social engagement, thereby affecting subjective wellbeing and quality of life. Consider patients with end-stage renal disease who must choose between different dialysis modalities (e.g., home-based peritoneal dialysis vs. in-center hemodialysis). When making this decision, they often focus on the immediate medical effectiveness and overlook the cumulative burden of repeated treatments. As a result, they systematically underestimate the inconvenience, discomfort, and lifestyle disruption that long-term dialysis entails. This affective forecasting bias can lead to an initial choice that seems acceptable at the outset but becomes progressively more burdensome over time. Consequently, patients may experience lower treatment satisfaction, reduced adherence, and even premature termination of therapy. Addressing such biases through better patient education, realistic simulation of daily treatment routines, or structured deliberation may improve the alignment between patients' expectations and their experience, thereby enhancing long-term satisfaction and health outcomes.

While affective forecasting is understood to rely on simulating future events, the specific cognitive mechanisms that determine the accuracy of these simulations in aging remain poorly understood. Identifying these mechanisms is, therefore, a critical step toward mitigating the public health impact of forecasting biases in the aging population. Therefore, this review first outlines the public health significance of affective forecasting biases in aging. It then examines theoretical explanations for these biases and the key boundary conditions that moderate age differences, before focusing on affective working memory as a critical yet underexplored cognitive mechanism. Finally, we present a study designed to clarify how AWM interacts with aging and event valence to shape forecasting accuracy, discussing the implications for promoting emotional health in later life.

Affective forecasting is defined as people's ability and process to predict their emotional reactions to future events (Wilson and Gilbert, 2003, 2005), and this process relies on the mental simulation of future events, which is often incomplete, leading to systematic biases—inaccuracies in estimating emotional outcomes (either overestimation or underestimation) (Wilson and Gilbert, 2013).

One explanation of such biases in simulation is the difference between participants‘ two-stage affective evaluation. In the prediction phase (i.e., the future event does not occur), individuals tend to focus on the fundamental characteristics of future events, whereas in the experience phase (i.e., the event occurs), they are immersed in richer, more subtle features of the event itself (Buechel et al., 2017). Another explanation is the difference in participants' ability to predict their future emotions across various dimensions (Lench et al., 2019). Specifically, people can accurately predict whether future events will make them feel good or bad, but are less accurate in predicting the affective duration and the affective impact of future events on emotions.

Though some previous studies believed that such affective forecasting bias stably emerged among both younger and older adults (Floerke et al., 2017; Kim et al., 2008; Nielsen et al., 2008; Pearman et al., 2010), some studies claimed that the forecasting pattern of both age groups is quite different (Barber et al., 2023; Lachman et al., 2008; Löckenhoff et al., 2011; Scheibe et al., 2011). The reason for this divergence has been summarized as differences in experimental design (Fernandez et al., 2025). More specifically, two experimental conditions that mainly lead to age-related differences in pattern are the attribute of future events itself (i.e., positive or negative) and the question framework (i.e., feelings in general or feelings for a focal event).

While valence does not eliminate forecasting biases *per se*, its interaction with aging reveals critical patterns. Previous studies have widely acknowledged that whether future events were positive or negative did not change the existence of affective forecasting bias itself (Wilson and Gilbert, 2003). However, when talking about the age-related differences in affective forecasting, it comes a little differently. While most affective forecasting literature tends to define overestimation in the prediction phase as the affective forecasting bias, a few researchers still find that underestimation occurs when the arousal characteristics of events in the prediction phase are not vivid. In contrast, experiences are quite vivid (Ebert and Meyvis, 2014). For example, people underestimated the impact of the aroma accompanying the taste of cookies on their experience, the level of happiness they experience when receiving smaller rewards and the level of unpleasantness they experience when watching negative videos (Lench et al., 2011; Buechel et al., 2017). And this is because there was a difference in affect-richness between prediction and experience under such conditions. If specific attributes of the to-be-predicted objects or events are internal perceptual clues that are relatively difficult for individuals to perceive and retrieve (e.g., the fragrance of cookies from the olfactory pathway), people can hardly make a mental simulation with such limited information about these attributes, leading to an underestimation. This implies that the patterns of affective forecasting bias might differ between younger and older adults. Because older adults often appear to prioritize more positive than the younger ones (i.e., positivity effects) (Barber and Kim, 2022), suggesting that there was an affect gap between the positive or negative future events based on such a preference division, and a potential difference between the older and younger adults might thus be anticipated. In fact, findings from Barber et al. (2023) provide some support for this assumption: their study found an age-related shift from positive to negative events. Specifically, older adults are not necessarily worse at predicting future emotions in all situations. Whether age differences emerge and how they do so depend on whether the emotional target is a global feeling or a specific event, as well as on the valence of the outcome and the emotion being predicted. This suggests that some of the age-related biases observed in previous studies may be more attributable to task specificity and individuals' focus of attention than to a general cognitive decline associated with aging itself. Therefore, when examining affective forecasting in aging, it is critical to separately consider positive and negative events, as their differential processing may underlie key age-related shifts in specific contexts, including health decision-making.

As another boundary condition reviewed by Fernandez et al. (2025), the role of the framework for the question in affective forecasting has also been widely examined among normal adults (Levine et al., 2012, 2013). This dimension, also referred to as focality in the literature, captures whether the affective forecasting question targets feelings tied to a concrete event vs. more diffuse, global affective states. Specifically, people showed poor affective forecasting when predicting feelings in general, but performed well when predicting only the focal events. This claim of Levine et al. differs from the previous conclusion of why the affective forecasting bias occurs (Wilson and Gilbert, 2013). It implies that focalism (the tendency to focus on salient features of an event) can also be seen as a promotion factor in such cases (feelings for a focal event but not in general) rather than only as a demotion factor for the accuracy of affective forecasts, conventionally. This protective role of the specific question framework can even weaken the effect of the age difference. For example, these age-related declines in accuracy found in Barber and Kim's (2022) study were attenuated when prompted to forecast feelings for the focal. This doesn't mean the non-existence of aging in affective forecasting, but rather should be interpreted as a need to reword the question's framework. Therefore, controlling for this variable is essential not only for methodological rigor but also for accurately isolating the unique contributions of aging and other cognitive mechanisms to forecasting bias.

Beyond these descriptive accounts and boundary conditions, identifying the specific cognitive mechanisms that determine simulation accuracy is crucial. Frank et al. (2021) proposed that affective working memory (AWM) plays such a role because this ability underlies actively maintaining and evaluating emotional feelings, a core ability in affective forecasting. To test this, they conducted a series of studies investigating whether individual differences in AWM are related to affective forecasting ability. In their study, the affect maintenance task proposed by Mikels et al. (2005) was adopted to measure the AWM; meanwhile, the analogous visual working memory task was also adopted to measure non-affective maintenance abilities to ensure that the AWM, rather than the whole working memory, affects affective forecasting (Mikels and Reuter-Lorenz, 2019; LeDoux and Brown, 2017). Specifically, in the affect maintenance task, participants were asked to maintain their emotional reaction to an emotional image (i.e., the intensity of their feelings) in mind over a delay (i.e., a brief interval). Then, they were required to compare their maintained emotional response to another one activated by the second image, and judge whether it was higher or lower. Their results show that, even when their AWM was higher and their working memory was relatively lower, participants tended to make more accurate forecasts. Therefore, Frank and his colleagues conclude that “...the results demonstrate a reliable and selective relationship between AWM and AF, suggesting that AWM is a separable working memory subsystem and an elemental capacity that contributes to the type of higher-order emotional processes involved in AF” (p. 67). Also, Frank et al. (2024) extended these findings to the real world in Experiment 4. The aim of this Experiment was to examine whether people could accurately predict their actual emotional responses to the outcome of the 2020 U.S. presidential election. Participants' affective forecasts and actual feelings were measured twice (i.e., before and after the election) to assess individual differences in affective forecasting accuracy and to analyze their relationship with working memory capacity. Researchers found that affective working memory performance significantly predicted people's affective forecasting accuracy for the election outcome. Moreover, this relationship was unique to affective working memory; cognitive working memory did not possess such predictive ability.

This raises a pivotal question for aging research: can older adults' relatively preserved AWM buffer against their broader declines in future-oriented cognition, thereby mitigating age-related forecasting biases? The answer is perhaps mixed. On one hand, previous studies have shown that though older adults showed typical decline in maintaining visual information, the ability to maintain emotional feelings was still maintained relatively well, which provides a prerequisite for its potential buffering effect (Mikels et al., 2005); On the other hand, apart from AWM (i.e., the ability to maintain emotional feelings), accurate affective forecasting also relies on one's capacity to mentally simulate future events. Or, it can be viewed as a product of future-oriented cognition (e.g., prediction or preview, etc.). Wilson and Gilbert have argued that when the content and context of the preview differ from those of the view, errors in emotional prediction occur (Gilbert and Wilson, 2009). The pervasive decline in cognitive working memory processes of older adults (Swanson, 2017) is no doubt at risk of expanding the discrepancy between the preview and the experience, particularly when the ability of future-oriented cognition generally presents an age-related decline as well (Kara-Yakoubian et al., 2023; Szpunar et al., 2014). This decline might lead us to expect age effects in affective forecasting despite the relatively well-preserved state of AWM. Indeed, if we take the attribute of future events itself (i.e., positive or negative) into consideration, the buffering effect of AWM on the aging of affective forecasting would definitely be more complicated, due to the positivity effects we mentioned above. As in Barber et al.'s (2023) study, the forecasting pattern might differ for younger and older adults, even with the benefit of AWM.

To address these questions and clarify the mixed potential of AWM, we first explore the age-related shifts in AWM; Then, we examine whether AWM affects participants' affective forecasting as well as its buffering effect on the aging of affective forecasting; Besides, we also contrast the pattern differences among positive or negative events to reexamine the potential positivity effects in older adults' affective reactions. In service of this goal, we replicated and expanded the experimental task adapted from Frank et al. (2021). The original task asks pairs to engage in a 60-min activity involving an affective forecasting task, an affect maintenance task, and an analogous visual working memory task to measure participants' abilities of affective forecasting, affective working memory, and working memory in an experimental context. For the present study, as we included some older adult participants, a shorter (30-min) version of the activity was executed to reduce cognitive load. Also, the measurement of emotional intelligence was excluded for the same reason, as it was deemed irrelevant to the research purpose. Importantly, the framework for affective forecasting in this study was limited to focal events to avoid confusion and interference.

With these in mind, our reasoning leads to three straightforward assumptions:

Hypothesis 1: We predicted that age differences in forecasting bias will be moderated by event valence. Specifically, we predicted that: (1a) relative to younger, older adults would be more positive in the total emotional responses; (1b) the bias (as in previous findings, underestimation in positive ones and overestimation in negative ones) would be thus moderated by the age group. That is, compared to the younger adults, older adults would predict more accurately due to the increase of their experienced emotional responses, which made the bias less; (1c) but this effect only existed when the materials were positive (due to the pattern of age effect was positivity effects).

Hypothesis 2: we predicted that: (2a) there would be a decline of AWM in the older adults, relative to younger, older adults would have a worse AWM; (2b) this decline might affect participants' total emotional responses, those who lacked abilities in maintaining their emotional reaction to specific events would have a bad performance on emotional tasks.

Hypothesis 3: As such, AWM was expected to have a mixed interaction with the aging in affective forecasting. We predicted that: (3a) AWM was positively correlated with the accuracy in affective forecasting; (3b) which, in turn, led to a difference between the older and younger adults. Specifically, older adults would benefit more from a higher AWM for their potential deficits, relative to younger adults.

Hypothesis 4: Beyond that, we predicted that AWM would not independently predict participants‘ affective forecasting. Specifically, participants' abilities in the presentation of such events, which are examined as visual imagery abilities, could also predict how accurately people anticipate their feelings about future events.

## 2 Methods

### 2.1 Transparency and openness

This research was approved by the Northeast Normal University Ethics Committee (project number: 2021043) as part of a larger protocol (Affective Forecasting Bias and its cognitive mechanism), and the following procedure was conducted in line with the relevant guidelines and regulations. There were no preregistrations. Data and analytic methods that support the findings of this study are available from the corresponding author upon request.

### 2.2 Design

This study adopted a 2 (age group: younger adults vs. older adults) ^*^ 2 (AWM group: high vs. low) ^*^ 2 (affective reaction phase: forecast vs. experience) mixed design. Specifically, only the affective reaction phase was a within variable, meaning that participants must complete the assessment task at two time points. The dependent variable is participants' emotional response (the intensity of affective reaction) to the specific events/pictures rather than to the general situation. Further, the visual representation ability and current emotional state were controlled as additional variables to be excluded for interference, and will not be discussed further.

### 2.3 Participants

A priori power analysis indicated that, to achieve a medium effect (80% power) with the criterion of statistical significance (f = 0.25), 68 participants were required for a repeated-measures analysis (Faul et al., 2007). Thus, we recruited 78 participants at first. However, following a priori exclusion criteria, 10 outliers were removed for poor performance (i.e., no reactions or reported unable to understand the program after the study). Thus, the final sample comprised 68 participants (34 older adults and 34 younger adults).

To be eligible, participants for both age groups were required to be right-handed and native Chinese Mandarin speakers; also, all participants were included only when they were physically and psychologically healthy (self-reported with no color blindness or color weakness, no diagnosed serious physical, and no diagnosed mental or psychological illness); Further, all participants were able to independently complete experimental procedures and make evaluations. In the older adults group, participants were required to be over 65 years old (64.71% female, *M*_*age*_ = 73.56, *SD*_*age*_= 5.40), while participants in the younger adults group were college students (47.06% female, *M*_*age*_ = 20.79, and *SD*_*age*_= 1.49). Participants were recruited through convenience sampling. Specifically, elderly subjects were recruited from the community near the university campus, and student subjects were recruited from China Medical University. All participants provided informed consent and received monetary compensation of 20 (RMB).

### 2.4 Procedure and measures

In general, participants completed two face-to-face testing sessions spanning 1 week at the China Medical University. All participants completed the affective forecasting task and assessments of emotion-maintenance and visual imagery abilities. All tasks were created in E-prime 3.0.

In Session 1, participants needed to complete the emotion-maintenance and affective forecasting tasks; they were also asked to complete the Vividness of Visual Imagery Questionnaire (VVIQ) and the Positive and Negative Affect Scale. In Session 2, participants were asked only to complete the emotion-maintenance task, the corresponding intensity rating tasks, and the affective forecasting task. For all participants, Session 2 took place 1 week after Session 1. The order of two tasks and description-based forecasting delay were intended to match the procedures used by Frank et al. (2021).

**Figure 1:**
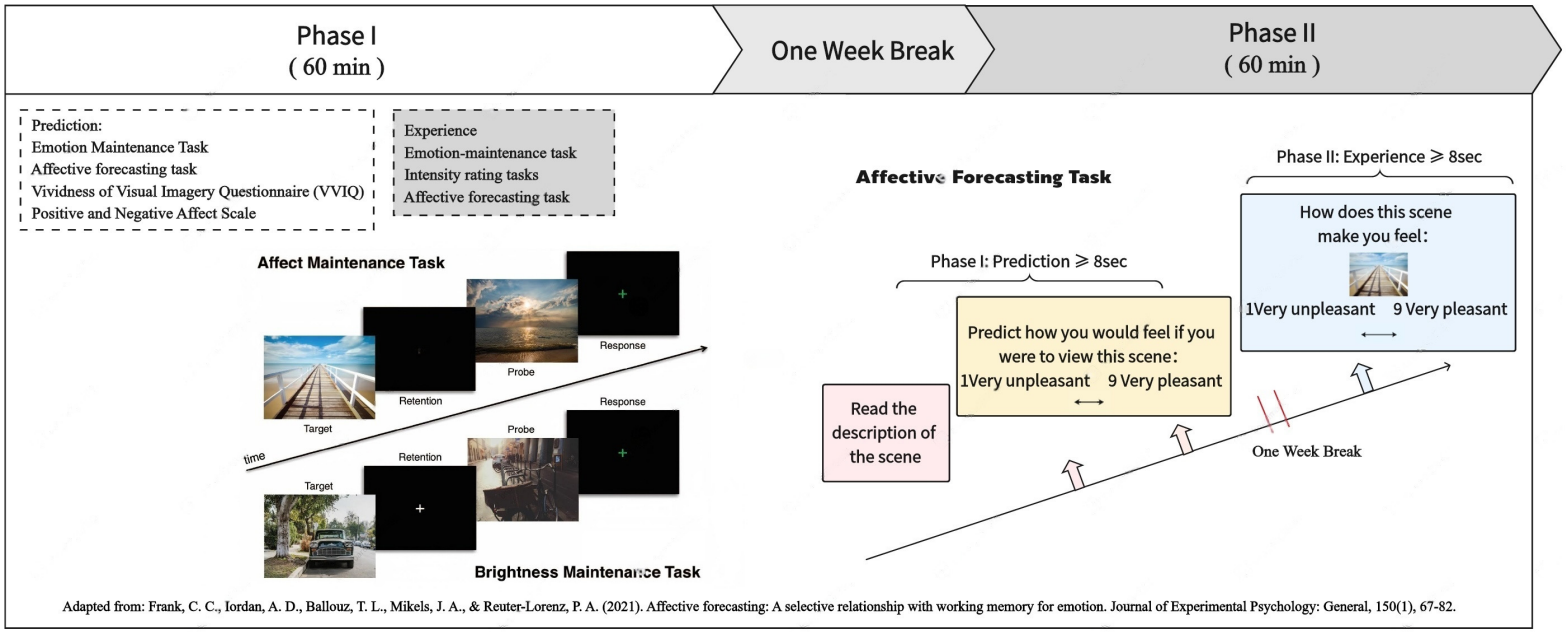
Schematic overview of the experimental procedure. The study consisted of two phases (60 min each). Phase I included an Emotion Maintenance Task, an Affective Forecasting Task, the Vividness of Visual Imagery Questionnaire (VVIQ), and the Positive and Negative Affect Scale (PANAS). Phase II comprised an Emotion Maintenance Task with an 8-second experience phase, intensity rating tasks, and an Affective Forecasting Task with scene descriptions followed by ratings of predicted and experienced pleasantness (1 = Very unpleasant to 9 = Very pleasant). The procedure was adapted from Frank et al. (2021), with two modifications for older adult participants: **(a)** the overall protocol was shortened to reduce cognitive load, and **(b)** filler measures (Brightness Maintenance Task and Emotional Intelligence Measures from the original study) were excluded to align with the research objectives. Adapted from: Frank et al. (2021).

However, there were still two little but important changes compared to the study of Frank et al. (2021). First, because we adopted participants who were older adults, the whole experiment had to be a shorter version based on pilot work, intended to reduce cognitive load so that participants in the older adults group were more likely to focus on the procedure. Second, some filler measurements, such as the Brightness Maintenance task and the Emotional Intelligence Measures in the original study, were excluded because of the research objective and synthesis (see more details in Figure 1).

#### 2.4.1 Emotion maintenance task

The emotion maintenance task was adapted from Mikels et al.'s (2005; 2008) previously used measure of AWM (Broome et al., 2012). For each trial, participants were first presented with a single emotional picture (5s) and instructed to maintain the feelings elicited by it. After a retention interval (3s), participants were then presented with the second emotional picture (5s). Finally, a green cross appeared, representing the response phase, and participants were instructed to report whether the second image had higher or lower emotional intensity than the first (by pressing either a key labeled H for higher or L for lower).

There were 24 trials in total, and each trial consisted of a pair of images selected from the International Affective Picture System (IAPS) (Bradley and Lang, 2017; Branco et al., 2023), with matching valence (12 positive and 12 negative trials). Specifically, these pairs of images were generated from a set of 106 images (53 positive and 53 negative).

The accuracy score was individualized based on each participant's ratings of emotional intensity in the emotional intensity rating task. Specifically, if the participant's relative-intensity ratings for the members of each pair matched the response on the affect maintenance task, it was recorded as correct. If they were equal, these trials were excluded from the calculation of that participant's accuracy scores. For each participant, accuracy scores were calculated as the number of correct trials divided by the total number of trials. Only when the accuracy score was over 50% would it suggest that participants were hardly guessing or otherwise not following instructions (this rule applied to all participants).

Further, the accuracy score was used to allocate participants into either the high AWM group (upper 50%) or the low AWM group (lower 50%). Due to the differences across age groups, the manipulation check was reported individually: for older adults, those in the high score group (*M* = 0.71, *SD* = 0.05) showed significantly more correct rates than the low one (*M* = 0.63, *SD* = 0.05), *t*_(32)_ = 5.02, *p* < 0.001; also, for younger adults, those in the high score group (*M* = 0.78, *SD* = 0.06) showed significantly more correct rates than the low one (*M* = 0.67, *SD* = 0.05), *t*_(32)_ = 6.37, *p* < 0.001.

#### 2.4.2 Affective forecasting task

The affective forecasting task has previously been used to assess affective forecasting accuracy (Frank et al., 2021, 2024), and participants were instructed to either predict or experience in the two-phase task.

During the first session (the prediction phase), participants were presented with a scene description and asked to imagine it. Then, they were asked to predict how they would feel if they viewed the actual image and rate that feeling on a visual analog scale ranging from very unpleasant to very pleasant. During the 1-week later Session 2 (the experience phase), participants were presented with the actual images matched to each description and asked to rate how each image made them really feel using the same scale.

There were 10 images in total, each selected from the International Affective Picture System (IAPS), which has matching valence (five positive and five negative). Specifically, descriptions and images were displayed on a computer screen, and the order of stimuli was randomized for each participant independently for Phase I and Phase II. Also, the rating scores were recorded from participants' mouse clicks anywhere along the scale.

To emphasize, we focused not only on magnitude (i.e., the deviation score) but also on the direction of affective forecasting bias (i.e., overestimation vs. underestimation), which differs from previous studies (Frank et al., 2021). Therefore, AF prediction-accuracy scores were calculated as the rating score, and the bias was reflected in the effect of the affective reaction phase (forecast vs. experience) on them (Wootton et al., 2025; Dillard et al., 2020).

#### 2.4.3 Positive and negative affect schedule

The Positive and Negative Affect Scale (PANAS) was used to assess participants' current emotional states (i.e., seen as an additional variable to be controlled and will not be discussed further), which consists of 20 words that describe feelings and emotions was employed with positive items α = 0.85, negative items α = 0.90 in previous research (Watson et al., 1988). For each word, participants were instructed to rate the extent to which they feel that way right now, using a 5-point Likert scale from 1 (*not at all*) to 5 (*extremely*).

#### 2.4.4 Vividness of visual imagery questionnaire

The visual imagery was assessed with the Vividness of Visual Imagery Questionnaire (VVIQ; Marks, 1973), as used in previous studies (Frank et al., 2021, 2024). Participants were asked to answer four questions about each of four imagined scenes, and to rate vividness on a 5-point Likert scale from *1* (*no image at all, you know nothing but your thought of the object*) to 5 (*perfectly clear and as vivid as normal vision*). The sum of all 16 item scores was the composite VVIQ score. Specifically, a higher score indicated a better imaging capability^1^.

## 3 Results

First, we computed Pearson's *r* correlations among the main variables (i.e., VVIQ, both PANAS types, the affective forecasting task, accuracy in the emotional maintenance task, and age) to replicate correlations reported in previous research (Frank et al., 2021, 2024).

As predicted, except for the VVIQ [*r*_(66)_ = −0.08, *p* = 0.529], there were significantly relationships between participants' age and every of the accuracy in the emotional maintenance task [*r*_(66)_ = −0.35, *p* = 0.003], the positive PANAS [*r*_(66)_ = 0.40, *p* = 0.001], the negative PANAS [*r*_(66)_ = −0.24, *p* = 0.045], and the positive affective forecasting task [*r*_(66)_ = −0.49, *p* < 0.001], which indicated that the aging effect we assumed did exist in this study generally.

**Figure 2:**
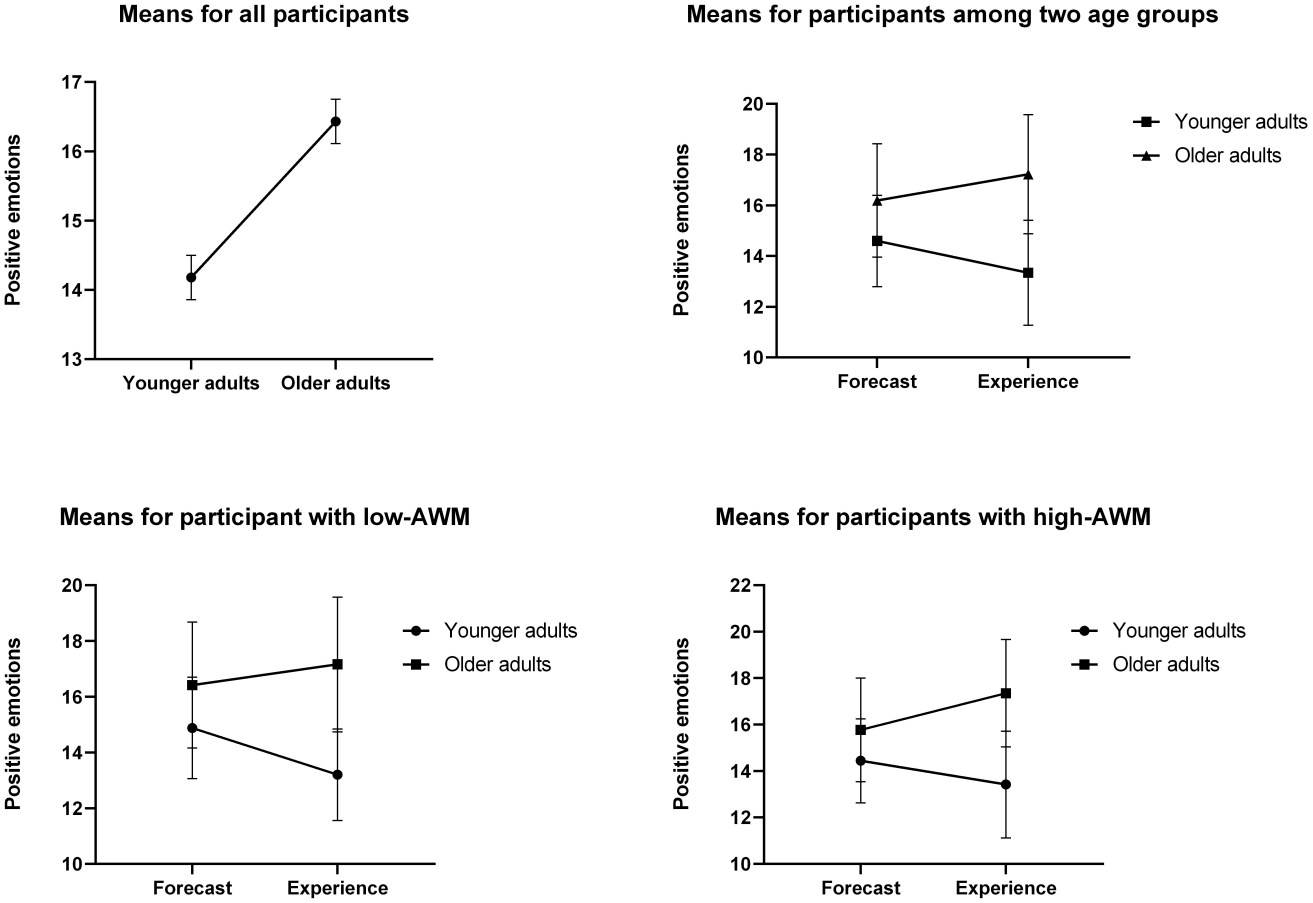
Mean ratings (±SD) of predicted and experienced positive emotions across participant groups. Data are presented as means with error bars representing standard deviations for four subgroup comparisons: **(A)** all participants, **(B)** younger vs. older adults, **(C)** participants with low AWM vs. high AWM, and **(D)** younger vs. older adults within each AWM subgroup. The figure provides a descriptive summary of the raw data. Inferential statistical results are reported in the main text (see Results section).

Further, the VVIQ was only found significantly related to the positive PANAS [*r*_(66)_ = 0.34, *p* = 0.005], meanwhile the accuracy in the emotional maintenance task was also found significantly related to the positive PANAS [*r*_(66)_ = −0.30, *p* = 0.014], which indicated that the positivity effects might exist in this study as well (for all descriptive statistics in this section, see Table 1). Therefore, the analyses focused on the dependent variable (i.e., affective forecasts and experiences) in positive and negative emotions, which were presented separately after controlling for gender, VVIQ, and both types of PANAS as covariates. Descriptive statistics for the main variables are summarized in Figure 1, which displays the mean ratings (± SD) of predicted and experienced positive emotions for all participants, by age group (younger vs. older), by AWM level (low vs. high), and by the combination of age and AWM groups. As shown in Figure 2, both younger and older adults exhibited a discrepancy between forecasts and experiences, with the direction of bias appearing to differ across groups.

**Table 1:** Descriptive statistics and correlations of main variables in the general results (*n* = 68).

Variable	*M* ±*SD*	1	2	3	4	5	6	7
1. Age	47.18 ± 26.87	1						
2. AWM	69.63 ± 7.47	−0.35^**^	1					
3. VVIQ	59.72 ± 9.63	−0.08	0.03	1				
4. PANAS-positive	32.29 ± 8.29	0.40^**^	−0.30^*^	0.34^**^	1			
5. PANAS-negative	19.19 ± 6.37	−0.24^*^	0.05	−0.21	0.09	1		
6. AFB-positive	0.11 ± 2.41	−0.49^***^	0.06	0.04	−0.02	0.19	1	
7. AFB-negative	0.39 ± 2.058	−0.10	−0.11	−0.06	−0.12	0.04	0.15	1

^*^p < 0.05, ^**^p < 0.01, and ^***^p < 0.001; 1. Age, 2. AWM, the scores in the affective working memory task; 3. VVIQ, the scores in the Vividness of visual imagery questionnaire; 4. PANAS-positive, the positive scores in the positive and negative affect schedule; 5. PANAS-negative, the negative scores in the positive and negative affect schedule; 6. AFB-positive, the difference scores in the affective forecasting task for positive images; 7. AFB-negative, the difference scores in the affective forecasting task for negative images.

### 3.1 Positive emotions

Then, all continuous predictors were mean-centered before analysis. Linear mixed models (LMMs) were fitted using the lmer function from the lme4 package (Bates et al., 2015), with restricted maximum likelihood (REML = FALSE) for model comparisons. Satterthwaite's approximation was used for degrees of freedom and *p*-values. Random intercepts for participants were included to account for repeated measures (two time points: affective forecasts and experiences). To assess the significant interaction in the positive-affect model, we computed cluster-bootstrap confidence intervals (1,000 resamples, resampling at the participant level) for the simple effects to obtain robust interval estimates.

Model comparisons are summarized in Table 2. For positive affect, the model including all two-way interactions (AWM × age, AWM × affective reaction phase, and age × affective reaction phase) provided a significantly better fit than the main-effects model, χ^2^_(3)_ = 20.30, *p* < 0.001. Adding the three-way interaction did not further improve model fit, χ^2^_(1)_ = 0.05, *p* = 0.825. Therefore, the two-way interaction model was retained.

**Table 2:** Model comparisons for positive and negative affect (*n* = 68).

Affect	Model	AIC	BIC	logLik	χ^2^	*df*	*p*
**Positive**	Null	622.21	630.95	−308.11	–	–	–
	Controls	602.63	623.02	−294.32	27.58	4	< 0.001
	Main effects	585.14	614.27	−282.57	23.49	3	< 0.001
	**All two-way interactions**	**570.84**	**608.71**	**−272.42**	**20.30**	**3**	**< 0.001**
	Three-way interaction	572.79	613.57	−272.40	0.05	1	0.825
**Negative**	Null	579.23	587.97	−286.62	–	–	–
	Controls	570.25	590.64	−278.12	16.99	4	0.002
	**Main effects**	**566.91**	**596.03**	**−273.45**	**9.34**	**3**	**0.025**
	All two-way interactions	571.51	609.37	−272.75	1.40	3	0.705
	Three-way interaction	573.50	614.28	−272.75	0.002	1	0.961

The interaction model included all main effects plus AWM × age, AWM × affective reaction phase, and age × affective reaction phase. The selected optimal model for each affect is shown in bold.

Table 3 provides more details on the fixed effects. The critical age × affective reaction phase interaction was significant [*b* = 2.33, *SE* = 0.51, 95% Wald CI [1.33, 3.33], *t*_(68)_ = 4.59, *p* < 0.001], whereas neither AWM × affective reaction phase (*b* = 0.03, *SE* = 0.03, 95% CI [−0.02, 0.08], *p* = 0.290) nor AWM × age (*b* = 0.05, *SE* = 0.04, 95% CI [−0.03, 0.13], *p* = 0.211) reached significance.

**Table 3:** Fixed effects of the optimal model for positive affect (two-way interaction model, *n* = 68).

Predictor	*b*	*SE*	95% Wald CI	*t*	*p*
Intercept	14.78	0.39	[14.01, 15.55]	37.42	< 0.001
Gender (2 vs. 1)	0.18	0.39	[−0.58, 0.94]	0.45	0.653
VVIQ (centered)	0.07	0.02	[0.02, 0.11]	3.06	0.003
PANAS-P (centered)	0.05	0.03	[−0.00, 0.11]	1.83	0.072
PANAS-N (centered)	−0.03	0.03	[−0.10, 0.03]	−0.93	0.354
AWM (centered)	−0.01	0.03	[−0.07, 0.05]	−0.35	0.727
Age (2 vs. 1)	1.12	0.51	[0.12, 2.12]	2.20	0.030
Affective reaction phase (Time2 vs. Time1)	−1.27	0.36	[−1.98, −0.57]	−3.55	< 0.001
AWM × Age	0.05	0.04	[−0.03, 0.13]	1.26	0.211
**AWM** **×Affective reaction phase**	**0.03**	**0.03**	**[−0.02, 0.08]**	**1.07**	**0.290**

Bootstrap CI based on 1,000 cluster-resamples (participant-level resampling).

Simple-effect analyses revealed that for the younger participants, positive affect decreased from Time 1 (forecast) to Time 2 (experience), estimate = −1.27, *SE* = 0.36, *t*_(71.1)_ = −3.47, and *p* < 0.001. For the older participants, positive affect increased from Time 1 to Time 2, estimate = 1.06, *SE* = 0.36, *t*_(71.1)_ = 2.88, and *p* = 0.005. Cluster-bootstrap 95% confidence intervals (1,000 resamples, participant-level resampling) for these differences were [−1.71, −0.85] for the younger group and [0.43, 1.71] for the older group, confirming the robustness of the cross-over interaction.

Further, VVIQ was a significant positive predictor (*b* = 0.07, *SE* = 0.02, and *p* = 0.003), and PANAS-P was marginally associated (*b* = 0.05, *SE* = 0.03, and *p* = 0.072). Neither gender nor PANAS-N nor AWM (main effect) reached significance (all *ps* > 0.35).

### 3.2 Negative emotions

For negative affect, model comparisons (Table 2) indicated that the main-effects model provided the best fit (AIC = 566.91). Adding two-way interactions did not improve model fit, χ^2^_(3)_ = 1.40, *p* = 0.705, and the three-way interaction was also nonsignificant, χ^2^_(1)_ = 0.002, *p* = 0.961. Therefore, the main-effects model was retained (see Table 4).

**Table 4:** Fixed effects of the optimal model for negative affect (main-effects model, *n* = 68).

Predictor	*b*	*SE*	95% Wald CI	*t*	*p*
Intercept	15.38	0.38	[14.63, 16.12]	40.43	< 0.001
Gender (2 vs. 1)	0.49	0.39	[−0.29, 1.26]	1.24	0.221
VVIQ (centered)	0.00	0.02	[−0.04, 0.05]	0.19	0.849
PANAS-P (centered)	0.05	0.03	[−0.01, 0.11]	1.70	0.093
**PANAS-N (centered)**	**−0.12**	**0.03**	**[−0.18**, **−0.05]**	**−3.51**	**< 0.001**
**AWM (centered)**	**0.05**	**0.02**	**[0.01, 0.09]**	**2.41**	**0.019**
Age (2 vs. 1)	0.54	0.45	[−0.34, 1.43]	1.20	0.234
Affective reaction phase (Time2 vs. Time1)	−0.39	0.25	[−0.88, 0.11]	−1.54	0.128

Significant effects (p < 0.05) are in bold.

PANAS-N was a significant negative predictor (*b* = −0.12, *SE* = 0.03, 95% CI [−0.18, −0.05], and *p* < 0.001): higher PANAS-N was associated with lower predicted negative affect. AWM showed a significant positive effect (*b* = 0.05, *SE* = 0.02, 95% CI [0.01, 0.09], and *p* = 0.019). No other effects were significant (all *ps* > 0.09).

To find more details, we further conducted a correlation analysis of participants' two-phase responses to negative emotions and these main variables (see Table 5). The results revealed that the relationship between age and the affective experiences was significant [*r*_(66)_ = 0.27, *p* = 0.026], indicating a potential age effect. Meanwhile, a weak correlation was found between affective forecasts and experiences [*r*_(66)_ = 0.36, *p* < 0.01], whereas a medium but stronger correlation was found between the experiences of positive and negative images [*r*_(66)_ = 0.54, *p* < 0.001]. Interestingly, these results also excluded the possibility of affective forecasting bias. This suggests that for negative emotional materials, participants' reactions in the prediction and experience phase might be too consistent (i.e., the negative materials are with a high information availability, which makes participants too sensitive to the negative emotions), which leads to the disappearance of the effect of the affective reaction phase. In fact, the significant relationship between current emotion states (measured as the negative PANAS) and participants' reactions in the prediction [*r*_(66)_ = −0.35, *p* = 0.003] and experience phase [*r*_(66)_ = −0.35, *p* = 0.004] could also provide some support for this assumption, which we would discuss in more detail in the discussion.

**Table 5:** Descriptive statistics and correlations of main variables in the two valence results (*n* = 68).

Variable	1	2	3	4	5	6	7	8	9
1. Age	1								
2. AWM	–	1							
3. VVIQ	–	–	1						
4. PANAS-positive	–	–	–	1					
5. PANAS-negative	–	–	–	–	1				
6. AF-positive	0.35^**^	−0.17	0.45^**^	0.47^**^	−0.16	1			
7. AE-positive	0.66^**^	−0.18	0.30^*^	**0.35** ^**^	−0.27^*^	0.59^***^	1		
8. AF-negative	0.20	−0.02	0.16	0.12	−0.36^**^	0.20	0.25^*^	1	
9. AE-negative	**0.27** ^*^	0.08	0.19	0.21	**−0.35** ^**^	0.21	**0.54** ^***^	**0.36** ^**^	1

^*^p < 0.05, ^**^p < 0.01, and ^***^p < 0.001; 1. Age, 2. AWM, the scores in the affective working memory task; 3. VVIQ, the scores in the vividness of visual imagery questionnaire; 4. PANAS-positive, the positive scores in the positive and negative affect schedule; 5. PANAS-negative, the negative scores in the positive and negative affect schedule; 6. AF-positive, the scores in the affective forecasting phase for positive images; 7. AE-positive, the scores in the affective experience phase for positive images; 8. AF-negative, the scores in the affective forecasting phase for negative images; 9. AE-negative, the scores in the affective experience phase for negative images.

Further, A paired-samples *t*-test showed that participants' arousal ratings for negative materials (*M* = 72.40, *SD* = 9.65) were significantly higher than those for positive materials (*M* = 67.63, *SD* = 9.67), *t*_(67)_ = −3.16, *p* = 0.002, Cohen's *d* = −0.38. The 95% confidence interval for the mean difference ranged from −7.78 to −1.75. These results indicate an asymmetric pattern of arousal between positive and negative stimuli, with negative materials eliciting stronger arousal than positive materials.

### 3.3 Exploratory analyses

To address the reviewers' comments and to further examine the robustness of our findings, we conducted a series of exploratory analyses. These analyses were not pre-registered and should therefore be interpreted with caution.

First, to directly test whether the positivity effect (i.e., older adults' relatively higher positive affect) is supported by our data, we fitted a linear mixed model with Age Group (Younger vs. Older), Valence (Positive vs. Negative), and Time (Time1 vs. Time2) as fixed factors, together with the covariates (Gender, VVIQ, PANAS-P, PANAS-N, and AWM) and a random intercept for participants. The critical Age Group × Valence interaction was significant, *F*_(1, 204)_ = 15.01, *p* < 0.001, partial η^2^ = 0.07. Simple-effect analyses revealed that older adults reported significantly higher positive affect than younger adults (estimated difference = −2.24, *SE* = 0.41, *t*_(147)_ = −5.49, and *p* < 0.001), whereas no age difference emerged for negative affect [estimated difference = −0.57, *SE* = 0.41, *t*_(147)_ = −1.39, and *p* = 0.165]. This pattern is consistent with a positivity effect. The three-way interaction Age Group × Valence × Time was also significant, *F*_(1, 204)_ = 4.41, *p* = 0.037, suggesting that the positivity effect may differ between the forecasting and the experience phase.

Second, we speculated that VVIQ might primarily influence the forecasting phase, whereas AWM might primarily influence the experience phase. To test these possibilities, we added the interactions VVIQ × Time to the positive-affect model and AWM × Time to the negative-affect model. For positive affect, the VVIQ × Time interaction was not significant, *F*_(1, 68)_ = 0.13, *p* = 0.723. For negative affect, the AWM × Time interaction was also not significant, *F*_(1, 68)_ = 0.21, *p* = 0.652. Furthermore, the three-way interaction VVIQ × Age Group × Time (tested in positive affect) did not reach significance, *F*_(1, 68)_ = 0.03, *p* = 0.874. Thus, although VVIQ and AM accuracy showed significant main effects, there was no evidence that their effects differed between the forecasting and the experience phases.

## 4 Discussion

Affective forecasting is integral to the health-related decisions of older adults, guiding choices from medical treatment to social engagement (Bosch et al., 2021; Karl et al., 2021). This study investigated the accuracy of these forecasts across adulthood and the potential moderating role of affective working memory (AWM). Given that previous research has shown that affective working memory is positively related to affective forecasting (Frank et al., 2021, 2024), our key finding reveals a divergent pattern of bias: older adults tended to underestimate future positive emotions, whereas younger adults overestimated them. This divergence underscores that affective forecasting bias in aging is not merely a matter of degree but of direction, with distinct implications for understanding and supporting decision-making in later life.

### 4.1 The aging pattern in affective forecasting

Our results partially supported the hypotheses regarding age differences. Across our results, as in many previous findings, older adults generated (both in the prediction and experience phase) more positive emotions than younger adults overall (Hypothesis 1a), which indicated that age differences in affective reactions (i.e., positivity effects) were generally built (Wootton et al., 2025; Barber and Kim, 2022).

But interestingly, the pattern of age-related differences in affective forecasting does differ from our assumptions (Hypothesis 1b, 1c): on one hand, aligning with the results in some previous studies, older adults underestimated their positive emotions to the same extent (Barber et al., 2023); however, on the other hand, the younger adults overestimated their positive emotions. Meanwhile, none of these patterns (under- and overestimation) was found in negative emotions. It is worth emphasizing that overestimation in affective forecasting itself is a quite common finding, as documented in our Introduction part. What really deserves to be addressed is why there is diversity in bias direction among younger and older adults.

One explanation of this age-related difference in the pattern of affective forecasting is based on the imbalance in the change in aging of experience. As mentioned, underestimation will occur when the arousal characteristics of events in the prediction phase are not vivid, whereas experiences are quite vivid (Ebert and Meyvis, 2014). In fact, for those previous studies reporting comparable affective forecasting bias, which is the underestimation, this premise also works: for example, in the study of Wootton et al. (2025), participants in both age groups were provided with a hypothetical scenario of engaging in a 10-min “sharing tasks,” which definitely lacks vividness compared to the following activity with a female confederate in the experience phase. Specifically, the study recruited younger and older adults and asked them to forecast the positive and negative emotions they would experience during a brief face-to-face interaction with a social partner (a female confederate). After the interaction, participants also reported their actual emotional experience during the interaction. In the previous study cited by Wootton et al. (2025) as support for the same pattern of under- and overestimation, a similar social task was also adopted in a younger sample and created a less engaging prediction task than the activity in the real experience phase (Moore et al., 2019). This indicated that the underestimation is non-age-related and the outcome of a specific prediction task (less engaging and not vivid enough).

If we compare our results with this rule, the underestimation among older adults fits: descriptions of images are indeed less vivid than the experience of the image. The more critical question is why younger adults in our study did not show the same underestimation but rather overestimation. To address this, we turn to the role of affective working memory (AWM) and the evaluability of the materials (see Sections 4.2 and 4.3).

### 4.2 The aging of AWM and its effect on affective forecasting

Our exploratory analysis directly tested the Age × Valence interaction, which was significant [*F*_(1, 204)_ = 15.01, *p* < 0.001]. Simple effects showed that older adults reported significantly higher positive affect than younger adults (*p* < 0.001), but there was no age difference for negative affect (*p* = 0.165). This pattern provides clear evidence for the positivity effect in our sample. Moreover, the three-way Age × Valence × Time interaction (*p* = 0.037) suggests that this effect is stronger during the forecasting phase, possibly because older adults' positivity bias is more active when imagining future events than when directly experiencing them.

We also assumed aging in AWM (Hypothesis 2a) and predicted it would lead to a lower emotional reaction among older adults (Hypothesis 2b). Our empirical results support both arguments. Compared with younger adults, older adults' AWM would be lower, whereas those with higher AWM abilities tend to perceive their emotional responses as higher. These findings imply that older people have lower AWM abilities and are expected to experience lower emotional responses than younger people. This appears contradictory to our initial expectations regarding age and forecasting.

Moreover, we did not find an interaction effect between AWM and the affective reaction phase, indicating that AWM doesn't shape affective forecasting as we predicted (Hypothesis 3a). Also, we cannot find an age-related difference in such a relationship (Hypothesis 3b). Older adults with higher AWM did not receive greater benefits, though they had more potential deficits in AWM than younger adults. Contrary to previous findings (Frank et al., 2021, 2024), the AWM seems less effective in both older and younger participants' affective forecasting.

As a comparison, we also examined the relationship between AWM and the accuracy of affective forecasting in another way: we re-defined the affective forecasting bias as the conception of absolute accuracy (Coundouris et al., 2023; Wootton et al., 2025; Barber and Kim, 2022) rather than with the experience as the baseline feelings, to determine whether predictions represent relative changes in emotion. The correlation results again suggested AWM doesn't shape affective forecasting as we predicted (Hypothesis 3a). Taken together, although AWM showed the expected age-related decline, it did not significantly contribute to the observed age differences in forecasting bias pattern.

It should be noted that younger adults appear to score significantly higher than older adults on AWM, indicating a bimodal distribution of participant ages, which leads to biased correlations between other variables and age (the so-called barbell effect). Therefore, correlations calculated within age groups might prove more informative. Fortunately, our findings from further analysis of the effects of AWM and VVIQ, using a two-stage approach, provide another potential explanation.

### 4.3 The dual-process and information available difference in affective forecasting

Specifically, treating the prediction and experience phases as independent emotional reactions, we surprisingly found that the VVIQ was related only to the prediction, whereas the AWM was related only to the experience. More importantly, there was no aging effect of VVIQ in our results.

Based on the dual process perspective in affective forecasting (based on the cognitive-experiential self-theory, CEST) (Dunn et al., 2009), when individuals are required to perform emotion prediction tasks, the essence of their predictions requires the participation of advanced cognitive abilities, which the VVIQ represents in our studies; while the predicted objects, that is, emotional experiences or emotions in the future state, is the marker of the experiential system, which is affected by the AWM in the present study. Our empirical results support the idea that deficits corresponding to normal aging occur only in AWM rather than VVIQ, indicating that the emotion predictions for both age groups were relatively close.

Then, let us step back and evaluate where our results first take us: we found that older adults showed an affective forecasting bias as an underestimation (forecast < experience), while younger adults overestimated their future emotional states (forecast > experience). If the upper argument (where emotion predictions for both age groups were relatively close) is right, that is, the key to diverse directions of bias is due to the differences in the experience phase. Indeed, aging-related decline in AWM ability may fill this gap: older adults exhibited poorer AWM, which was thought to be critical for the experiential system. This impairment may lead them to rely more on online affective processing during the experience phase, resulting in a higher experienced positive affect than they had forecasted, thereby establishing the underestimation pattern (forecast < experience) for the older group.

The explanation within such a framework emphasizes two key factors: the dual-process system difference throughout the process of affective forecasting (as we just discussed) and the information availability of to-be-predicted events. The explanation of how information available affects the decision-making process can be found in the general evaluability theory (GET), proposed by Hsee and Zhang (2010). When a value (i.e., property or criterion) is more sensitive and accessible, people tend to base their decisions on it. And we have discussed, for the task used in our study, the materials in the prediction phase may have relatively low evaluability, relatively to some social tasks used in previous studies (Moore et al., 2019; Wootton et al., 2025); while failing to increase emotional reactions in the prediction phase might further expand the difference between the prediction and the experience, potentially exaggerating age differences in forecasting bias. In other words, we are reasonable to suspect that the stability of age differences in forecasting bias findings in this study or previous studies is limited. To further investigate the impact of normal aging on affective forecasts, future research could examine this by manipulating changes in available information (artificially increasing information availability) when responding to specific features of the event.

Indeed, forecasting research in the context of aging focuses heavily on forecasting rare events (whether positive, negative, or neutral), with important implications for older individuals' transformative decision-making. For example, in a medical decision-making context, older patients often lack sufficient information for a deliberative, well-documented decision. This is because patients are unable to reliably predict their future emotional responses and well-being in anticipation of a health condition (Bosch et al., 2021). Given this persistent inaccuracy, it is important to understand the manifestation of such a bias (i.e., overestimation or underestimation), as this is the first step toward narrowing the gap between individuals' predictions and actual experience. These complex age-related shifts might have many implications; for example, in our study, a stronger emotional reaction (whether negative or positive) doesn't necessarily indicate more sensitive feelings toward the targets of events, suggesting a potential risk rather than an advantage of specific normative shifts.

To examine this assumption, exploratory analyses were executed. However, our exploratory analyses (see Exploratory analyses Section) examined whether VVIQ and AWM interacted with the forecasting vs. experience phase. Neither the VVIQ × Time interaction in positive affect nor the AM accuracy × Time interaction in negative affect reached significance (both *p* > 0.65). Thus, we found no evidence that these individual-difference variables selectively influence one phase over the other. Instead, VVIQ showed a robust main effect on positive affect (overall), and AWM showed a main effect on negative affect (overall), as reported in the main analyses.

Therefore, the earlier speculation that VVIQ supports the cognitive system (prediction) while AWM supports the experiential system (experience) is not supported by our data. A more parsimonious interpretation is that both VVIQ and AWM contribute to general emotional processing, but their effects do not depend on whether the emotion is being forecasted or experienced.

Turning to the age-related bias pattern, the significant Age × Valence interaction indicates that older adults report higher positive affect than younger adults, whereas, no age difference is observed for negative affect. This pattern is consistent with the positivity effect observed in many aging studies. The additional Age × Valence × Time three-way interaction suggests that the positivity effect may be more pronounced during the forecasting phase than during the experience phase, a novel finding warranting replication.

### 4.4 Public health implications

This framework is directly relevant to health contexts in which older adults make forecasts with limited information. For instance, when predicting post-operative recovery (an affect-rich experience) based on a surgeon's description (often affect-poor), older adults may underestimate their future emotional challenges, as seen in our study. This could result in inadequate psychological preparation. Interventions could focus on providing more evaluable information (e.g., vivid descriptions or patient testimonials) to reduce forecasting errors, rather than relying solely on improving AWM.

## 5 Strengths and limitations

A key strength of this study is its novel investigation of age differences in affective forecasting within the effect of affective working memory. This led to the key finding of a reversal pattern in affective bias (over- vs. under-estimation) among younger and older adults, indicating that the positivity effect was not as constructive as in previous evidence (Wootton et al., 2025) to date to complement the older adults' emotional sensitivity deficits, and may instead depend on task characteristics such as information availability.

This was important because it implied that the aging in affective forecasting (Barber et al., 2023; Lachman et al., 2008) might be the outcome of task differences (i.e., differences in information availability) rather than a real age-related difference. Recent evidence that can somehow support our inference on the potential role of information availability has been put out: it was found that younger adults were more sensitive to differences in affect-rich than in affect-poor outcomes, whereas older adults still showed similar sensitivity to differences in affect-rich and affect-poor outcomes (Frank and Pachur, 2025). While individuals, whether younger or older, were both similarly more risk-averse and made worse quality decisions for affect-rich than for affect-poor problems, which accord with our inference on the role of information availability, for an absence of (or very small) age differences in their results, just like our results in the negative emotions.

Contrary to previous findings (Frank et al., 2021, 2024), AWM did not interact with the forecasting vs. experience phase; its effect was limited to a main effect on negative affect. Although, we offered a dual-process interpretation, exploratory analyses showed no evidence for the claimed dissociation between VVIQ-prediction / AWM-experience (both interactions with time were non-significant). Therefore, we have removed that speculation and now interpret VVIQ and AWM as general predictors of emotional processing rather than phase-specific mechanisms. Future research should recruit samples with a wider range of AWM abilities and manipulate information availability to further test these relationships.

Several limitations should be acknowledged. First, the sample size (*N* = 68) is modest, which reduces statistical power for detecting higher-order interactions (e.g., the three-way Age × Valence × Time interaction was significant but should be replicated). Second, the affective forecasting task comprised only five positive and five negative trials, which may limit the reliability of individual bias estimates. Third, the intensity ratings used to assess AWM accuracy were collected 1 week after the task, raising concerns about the stability of participants' emotional intensity ratings over time. However, prior work has shown acceptable test-retest reliability for similar tasks (Broome et al., 2012). Fourth, we did not formally match the arousal levels of positive and negative stimuli; the higher arousal of negative stimuli (confirmed by a *post-hoc* paired *t*-test) may partly explain the valence asymmetry in forecasting bias. Fifth, the use of standardized laboratory stimuli may limit generalizability to real-world, personally relevant health decisions. Future research should employ larger, more diverse samples, increase the number of trials, and experimentally control stimulus arousal to isolate valence effects. Translating these findings into ecologically valid health-decision contexts (e.g., chronic illness management, surgical recovery) remains a critical next step. Despite these limitations, the study provides the first direct evidence of an age-reversed bias pattern for positive affect and highlights the role of information evaluability in shaping affective forecasting accuracy.

## Data Availability

The raw data supporting the conclusions of this article will be made available by the authors, without undue reservation.

## Footnotes

1 Here, our measurement is based on a reversed five-point Likert scale compared to the original and common version of VVIQ, to reduce the cognitive load for older adults and make it easier for them to understand our experiment instruction.
